# Detailed Experimental Study of Ion Acceleration by Interaction of an Ultra-Short Intense Laser with an Underdense Plasma

**DOI:** 10.1038/srep31647

**Published:** 2016-08-17

**Authors:** S. Kahaly, F. Sylla, A. Lifschitz, A. Flacco, M. Veltcheva, V. Malka

**Affiliations:** 1 Laboratoire d’Optique Appliquée, Ecole Polytechnique, ENSTA, CNRS, UMR 7639, 91761 Palaiseau, France; 2 ELI-ALPS, ELI-Hu Nkft, Dugonics ter 13, Szeged 6720, Hungary; 3 SourceLAB SAS, 86 rue de Paris, F-91400 Orsay, France

## Abstract

Ion acceleration from intense (*Iλ*^2^ > 10^18^ Wcm^−2^ *μ*m^2^) laser-plasma interaction is experimentally studied within a wide range of He gas densities. Focusing an ultrashort pulse (duration 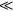 ion plasma period) on a newly designed submillimetric gas jet system, enabled us to inhibit total evacuation of electrons from the central propagation channel reducing the radial ion acceleration associated with *ponderomotive* Coulomb explosion, a mechanism predominant in the long pulse scenario. New ion acceleration mechanism have been unveiled in this regime leading to non-Maxwellian quasi monoenergetic features in the ion energy spectra. The emitted nonthermal ion bunches show a new scaling of the ion peak energy with plasma density. The scaling identified in this new regime differs from previously reported studies.

Ultrashort intense laser pulse, when focussed to extreme intensities, instantly ionises dense matter in the focal volume. Electrons and ions, with widely different inertia, respond and accelerate on different time scales during the interaction. The electrons can accelerate^1^ to relativistic energies leading to wide ranging effects including ultrafast^2^ as well as persistent^3^ magnetisation and emission of coherent XUV radiation^4^. Ions, being heavier, are accelerated as a result of the long timescale plasma dynamics involving the driving laser, the fast responding plasma electrons^5^ and the subsequent plasma electromagnetic and quasi-static field structures^6^,^7^ and thus constitute a very sensitive probe of the ensuing laser plasma process.

In the regime of a long pulse 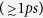 laser interaction with an underdense plasma (*n*_*e*_ < *n*_*c*_, where *n*_*e*_ and *n*_*c*_ are the electron and critical density respectively), ion acceleration has already been in experimental demonstrated. The associated physical mechanisms have also been identified for the *transverse* (Coulomb explosion^8^,^9^, collisionless shock expansion^10^) and *longitudinal* (sheath acceleration^11^, Magnetic Vortex Acceleration^12^) ion accelerations. Though the connection between longitudinal and transverse ion accelerations have not been addressed in detail, it is well known that the large laser pulse duration plays a decisive role in affecting ion motion during the pulse and in determining the subsequent ion energy distribution^8^,^9^. In Coulomb explosions, ions are accelerated by the ponderomotive charge separation over the driving pulse duration, leading to a Maxwellian ion spectrum in the case of long plasma jet^8^. However, when conditions for collisionless shock expansion are met, higher ion energies with a plateau-like spectrum are observed^10^. In all known cases of acceleration, ion energy monotonically scales up with the plasma density.

In contrast in the ultrashort pulse domain, when laser duration (*τ*) attains a substantially shorter value than the inverse ion plasma frequency (*τ*_*pi*_), the standard picture of ion acceleration depicting direct laser induced energy transfer within the ion response timescale becomes inadequate. Although this domain has been theoretically investigated^13^,^14^,^15^,^16^ and is of strong interest considering many ultrashort high repetition rate petawatt laser facilities coming online in the near future, there have been very few experimental studies^17^,^18^ and most importantly, the ion energy scaling with plasma density is unknown.

Here we present the first detailed experimental study of ultrashort laser-assisted ion acceleration from underdense He plasma in this new regime of interaction. We have performed experiments under *conditions* significantly different from previous studies where: (a) the laser duration is considerably shorter than the ion plasma period 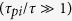 and (b) the interaction length (submillimetric gas jet length *L*) is comparable to the Rayleigh depth (*z*_*R*_) of the focussed laser beam in vacuum (*z*_*R*_/*L* ~ 1), reducing the multiple focusing within the plasma^9^. In a previous study^18^, we numerically tested the connection between longitudinal and transverse acceleration mechanisms with experimental evidences of ion acceleration occurring through a combination of sheath acceleration and Coulomb explosion of an ion filament formed around the laser axis. We now demonstrate that under these unexplored conditions, we observe ion acceleration that shows a new energy scaling with plasma density qualitatively different from previously reported scaling for the long pulse regime^9^,^10^. We also comment on the density dependent *relative abundance* of the two ion species detected in the interaction.

## Experimental set up and detection technique

The experiments have been carried out using the Salle Jaune Ti:S laser^19^ at the Laboratoire d’Optique Appliquée (pulse duration *τ* = 35 fs, wavelength *λ*_*μ*_ = 0.8 *μm* linearly-polarized pulses). Each pulse carrying average energy of 800 mJ is aberration corrected using adaptive optics and is focussed to a spot size of *d*_*μ*_ = 20 *μ*m (1/*e*^2^) on target with an *f*/10 off-axis Au coated parabolic reflector, delivering a peak intensity of 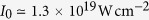 in vacuum. This leads to a dimensionless amplitude 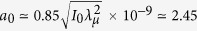 making the interaction with plasma electrons relativistic. The laser is focussed at the entry of a ~700 *μ*m (FWHM) hypergaussian He gas jet. The supersonic jet has a sharp edged hypergaussian neutral density profile with the peak density depending on the height from the nozzle output surface^20^. The plateau neutral atomic density is varied within the range ~[0.03 − 3.1] × 10^19^ cm^−3^ by changing either the backing pressure or the opening time of the valve^20^. Under these conditions, 
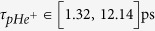
 and 
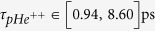
, with *τ*_*pi*_ = 2*π*/*ω*_*pi*_ = 2*π*(*m*_*i*_*ε*_0_/*n*_*i*_*Z*^2^*e*^2^)^1/2^, where *ω*_*pi*_, *m*_*i*_, *n*_*i*_ and *Ze* are plasma frequency, mass, density and charge respectively for the *i*^*th*^ ion species. This allows us to achieve *condition* (a) mentioned in the section above.

**Figure 1:**
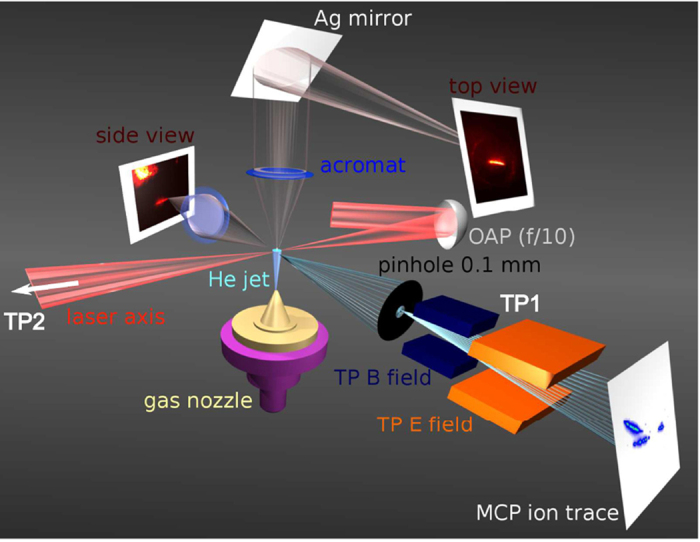
Experimental setup. The Thomson parabola (TP) 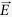 and 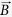 fields are parallel and aligned vertically upwards in the picture. A Thomson parabola ion spectrometer TP1 is placed at 80° from laser propagation direction (shown) with another identical one TP2 (not shown) is placed at 0° along the laser axis to look at ions accelerated in the forward direction. Top and side views imaging together locates the plasma channel position with respect to the gas nozzle.

An overview of the experimental setup is presented in Fig. 1. Simultaneous transverse imaging in the vertical plane (top view: using Thomson scattered *ω*) and in the horizontal plane (side view: using 2*ω* light emitted from plasma channel) coupled with five axis motion of the gas jet system (Fig. 1) allow precise alignment and complete control of the nozzle position with respect to the laser focal volume. The accelerated ions are collected through a 100-*μ*m pinhole (subtending 1.2 × 10^−8^ sr on the source) onto a Thomson parabola (TP) spectrometer (TP1) at 80° from laser propagation direction (Fig. 1), where quasi-monoenergetic ion bunches were obtained. The TP uses parallel electric and magnetic 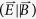 fields to disperse ions, depending on their charge-to-mass ratio and energy, finally tracing out parabolas on the detector plane as shown in Fig. 1. The tracks are detected with a 40-mm diameter phosphor coupled microchannel plate (MCP) and recorded on a 16-bit CCD camera. The pinhole image of 400 *μ*m (nozzle exit diameter) in the gas jet plane occupies ~7.5 pixels on the camera, thus giving almost a point image of the ion source. The MCP gain is adjusted so that whole dynamic range of the CCD is used whilst keeping the best signal-to-noise ratio (~50:1). Another identical system of TP ion spectrometer (TP2) is used to detect ions accelerated in the laser propagation direction. Under our detection scheme TP1 and TP2 have energy detection thresholds ~48 KeV for *He*^++^ and ~10 KeV for *He*^+^. The energy resolution attainable with the TP-MCP assembly is ~2 KeV.

## Experimental observation

### Focussing configuration

The laser power *P*_*L*_ used in our experiments is larger than the threshold of relativistic self focussing^21^ given by the critical power, 
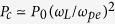
, where *P*_0_ = 17 GW and *ω*_*pe*_ is electron plasma frequency. *P*_*L*_/*P*_*c*_ ∈ [0.5, 50] for the range of plasma densities accessed in the experiments, ensuring that the *in situ* intensity is higher than in the vacuum giving access to the relativistic interaction regime. Additionally, the gas jet size is of the order of Rayleigh length *z*_*R*_ for our focusing conditions to avoid multiple focusing^9^ and thus satisfying condition (b) from the previous section.

**Figure 2:**
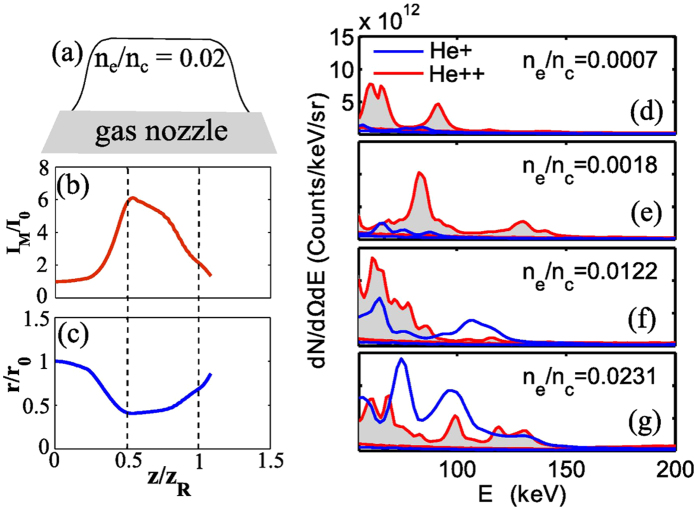
(**a**–**c**) Results of WAKE simulations. (**a**) Schematic showing longitudinal position of the hypergaussian gas plasma density profile relative to the simulation box. (**b**) Ratio of vacuum peak intensity *I*_0_ and peak intensity inside plasma *I*_*M*_ as a function of position *z* (*z*_*R*_ = Rayleigh length). (**c**) Ratio of laser beam size at the entrance *r*_0_ and inside plasma *r*. (**d**–**g**) Experimental measurements, showing variation in observed ion energy distributions of the two ionic species *He*^+^ and *He*^++^ as density is tuned from extremely underdense to mildly underdense regime. *n*_*c*_ is critical density given by 
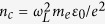
, where *ω*_*L*_, *m*_*e*_ and *e* are laser frequency, electron mass and electron charge respectively.

In order to verify these points, we performed simulations using the WAKE code^22^ under conditions similar to the experimental configuration. A typical example is shown in Fig. 2. Figure 2(b,c) shows the simulation results when the laser dimensionless field amplitude is 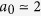 and interacts with the gas jet profile described in the previous section with plateau plasma electron density of *n*_*e*_ = 0.02 *n*_*c*_ (Fig. 2(a)). We observe in Fig. 2(b) that the vacuum laser peak intensity *I*_0_ is enhanced by a factor of 6 near 0.5 *z*_*R*_ due to self focusing inside the plasma. Figure 2(c) shows that the self focusing leads to corresponding reduction in laser beam size inside plasma and that there is no subsequent refocusing^9^ of the beam.

A simple analytical estimate of the higher field amplitude acquired by laser due to self-focusing can be made using stationary focusing condition^23^. This gives *a*_*SF*_ = 2(*P*_*L*_*n*_*e*_/*πP*_0_*n*_*c*_)^1/3^ while the self-focussed focal spot diameter is, 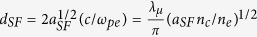. This analytical model slightly overestimates the absolute value of *a*_*SF*_ whilst underestimates *d*_*SF*_ when compared to the WAKE simulations values. The scaling of both *a*_*SF*_ and *d*_*SF*_ with plasma density *n*_*e*_ qualitatively corroborates the WAKE simulations.

### Energy distribution of accelerated ions

During the experiment, diagnostics TP1 and TP2 were simultaneously looking at ions accelerated in the transverse and the forward directions respectively as depicted in Fig. 1. Under our irradiation conditions and explored plasma densities. There was no detectable ion signal in TP2, i.e. no ions in the forward direction beyond the energy detection threshold was recorded. However, the TP1 ion traces show the existence of both *He*^+^ and *He*^++^ ion species for all experiemental conditions.

The observed spectra for energies below 50 KeV, where only *He*^+^ is accessible by the TP diagnostic due to its lower detection threshold for *He*^+^, show Maxwellian-like energy distribution^18^. In Fig. 2, we show typical *He*^+^ and *He*^++^ spectra between 50 KeV and 200 KeV recorded in the transverse direction for different values of density. Within this energy window and at lower plasma electron densities (Fig. 2(d,e)), the ion energy distribution shows striking features like quasi-monoenergetic peaks. These features gradually wash out and become less clear as plasma electron density is increased further (Fig. 2(f,g)).

Detailed realistic simulations using particle in cell Calder-Circ code^24^ for the exact experimental gas jet configuration and continued for sufficient time in order to track complete ion dynamics have previously allowed a qualitatively reproduction of the observed main ion spectral features and gain insight into the acceleration mechanisms^18^. No forward acceleration is observed in the simulation confirming experimental observation.

Figure 2 also captures the general relative tendencies shown by *He*^+^ and *He*^++^ energy distributions at different densities. We note that at very low densities (Fig. 2(d,e)) *He*^++^ population dominates *He*^+^ and this trend gradually reverses while increasing the density (Fig. 2(f,g)). This point will be addressed later.

### Scaling of ion cut off energy with plasma electron density

**Figure 3:**
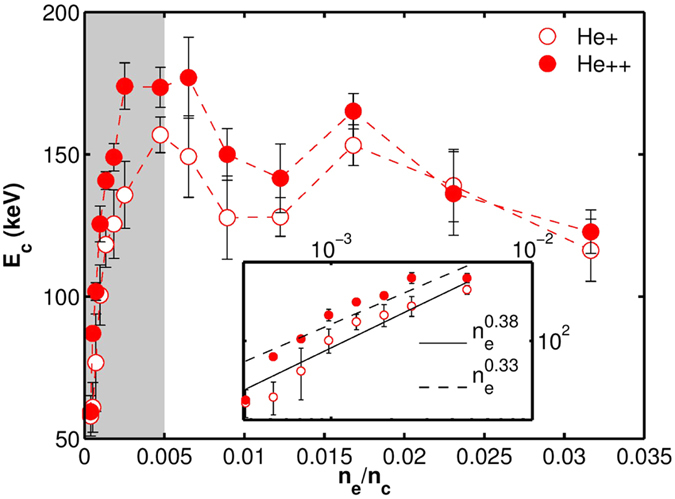
Cut off energy dependence on plasma electron density. The error bars represent standard deviation of the observed ion energy cut off obtained from different single shot ion energy spectrum collected under identical conditions. The inset replots the shaded region and shows power law fits to the variation.

Ion cut off energy *E*_*C*_ is defined as the highest energy of the ion track for which ion counts above the average background noise could be observed on the MCP (Fig. 1). Figure 3 shows in detail the variation of *E*_*C*_ with plasma electron density. We note that *E*_*C*_ of both the accelerated ion species show similar behaviour with changing *n*_*e*_. However the cut off ion energy is not monotonically increasing with density as observed by Wei *et al.*^10^ in their case. In our experiment, we managed to reach an interaction regime beyond the ones previously reported and crossing an optimum of density for the acceleration. Thus we observe two clearly distinct regions of interest: a very low density region (*n*_*e*_ ≤ 0.005 *n*_*c*_) where *E*_*C*_ increases sharply with density (from few keV to well above 100 keV, as shown in the shaded region in Fig. 3) and a slowly varying region where *E*_*C*_ appears to decreases slowly.

The inset shows that ion cut off energy for both the species scales in a similar way with density within the shaded region (for *He*^+^, 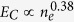 and for *He*^++^, 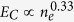). These results differ significantly from the scaling of 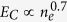 reported in reference^10^.

Preliminary simulations using a fully relativistic 3D PIC code, CALDER^25^, over short times (<1 *ps*) for comparable plasma density profiles at a few different density values indicate similar qualitative trend as observed in the experiments, i.e. an increase in *E*_*C*_ followed by saturation with increasing *n*_*e*_. A more quantitative estimate for identifying the ion energy scaling would need much longer simulation times, sufficient enough for capturing the complete ion dynamics, and a scan over many density points with the realistic plasma density profiles. This is computationally very expensive and at present beyond scope but would be a topic of future investigations.

### Relative population of detected *He* ^+^ and *He* ^++^ and effect of plasma electron density

Now we look at the relative abundances of the two accelerated ion species in our experiment. Firstly, Fig. 3 shows that both *He*^+^ and *He*^++^ have a very similar *E*_*C*_ values. This observation suggests that *He*^+^ and *He*^++^ are *not* accelerated from the interaction volume by an electrostatic, and thus charge dependent, acceleration mechanism. The laser channel is devoid of *He*^+^ during the interaction. The *in situ* laser peak intensity, which is many orders of magnitude higher than the field ionization threshold (*I* ≥ 10^16^ *W*/*cm*^2^) of *He*^+^, would completely ionise all helium gas throughout the interaction volume. With charge exchange effects, the observed *He*^+^ ions would most probably be accelerated *He*^++^ projectiles that captured electrons in elastic collision with background neutral *He* atoms rather than being accelerated by the fields in the plasma (one-electron-capture cross-section *σ*_*e*_ of the order of 10^−16^ cm^−2^ in the observed energy range^26^,^27^). This effect, as the laser-accelerated ions propagate out of the interaction region and reach the detector, strongly affects the measured spectral populations without significantly changing the ion energies. The phenomenon has been briefly suggested in refs 8,10 and now we will elaborate upon their arguements with further experimental evidences.

**Figure 4:**
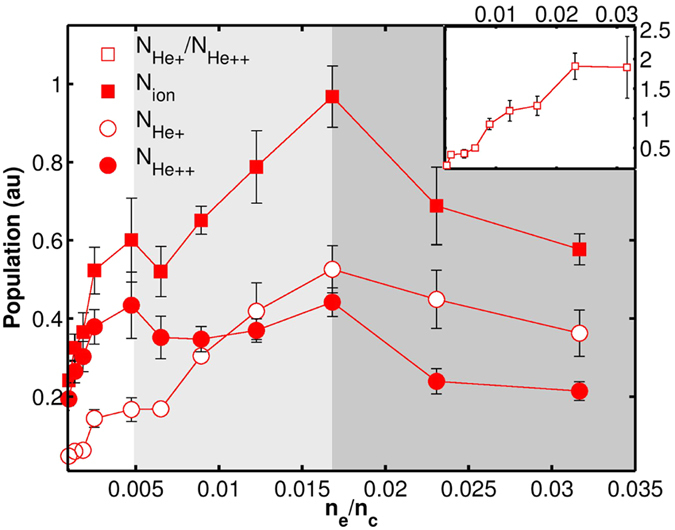
Variation of population of different accelerated ionic species with plasma electron density. The inset on the upper right corner shows the monotonic increase of relative population of detected *He*^+^ with respect to *He*^++^ with increase of density.

The evidence of charge exchange is clear in Fig. 4. 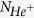 (resp. 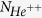) represents the total *He*^+^ (resp. *He*^++^) counts per unit solid angle on the detector integrated over the energy range [50–180] keV, i.e. 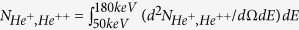. Each point on the graph is an average over 5 to 10 shots under identical conditions. 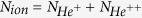 represents sum total of all the detected ions in this energy range per steradian. We identify and emphasize three distinctive regions in Fig. 4 (demarcated by the different shades): (I) the low density region upto 0.5% *n*_*c*_ (white) where *N*_*ion*_, 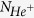 and 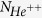 monotonically increase with density (same density range as the gray region of Fig. 3); (II) the mid density region (light gray) where *N*_*ion*_ increases, but the number of 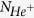 increases at the expense of 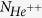 and (III) the high density region (dark gray) spanning from ~1.7% *n*_*c*_ to 3.2% *n*_*c*_, where the total number of ions reaching the detector decreases with density, indicating a less efficient acceleration and/or significant charge exchange and neutralization.

Region (I) corresponds in Fig. 2 to (d) and (e), where the abundance of *He*^++^ dominates over *He*^+^. Regions (II) and (III) correspond respectively to (f) and (g) in Fig. 2, where the abundance of *He*^+^ starts to dominate over the *He*^++^. We also note that in region (I), which coincides with the shaded region in Fig. 3, both the maximum accelerating field (*E*_*C*_) and total number ions in the accelerating volume (assuming cylindrically symmetric acceleration) are increasing with *n*_*e*_.

Finally, we can also plot 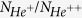, which represents the ratio of *He*^+^ to *He*^++^ counts (inset in Fig. 4), increasing almost monotonously with gas density throughout all the three identified gas density regimes and then seems to saturate. This observation is in good agreement with the fact that the increasing ambient gas density increases the probability of one-electron-capture by *He*^++^ ions and, at high densities, similarly affects the population of *He*^+^ ions.

## Discussion of the Results and Perspective

The experimental results presented in the previous section show that this regime of ion acceleration can lead to several new observations: (i) quasi monoenergetic features in the ion energy distribution; (ii) predominant ion acceleration in the near transverse direction; (iii) power law scaling followed by saturation-like behaviour of *E*_*C*_ with plasma density and (iv) evidence of conversion of accelerated *He*^++^ to *He*^+^ due to propagation through the gas density. Below we summarize parameters that play important role in ion acceleration process and then present a qualitative discussion of the above mentioned points.

### Parameters affecting ion acceleration in the new regime

The most important parameters for a given gas density profile that affect ion acceleration process from underdense plasma by influencing the relevant sub-processes are summarized in Table 1. For illustrative purposes we contrast our experimental parameter space with that of a previous study^10^ for which ion energy scaling data is available as discussed previously.

In reference^10^: *τ*_*pi*_/*τ* ~ 1, implying that ion dynamics within the pulse is important; 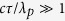 implying they are mainly within the self modulated wake field acceleration (SMWF)^28^ electron acceleration regime; *P*_*L*_/*P*_*c*_ values in combination with *τ*_*pi*_/*τ* imply a strongly relativistic regime where the long pulse would be self-focussed^29^ and evacuate the central channel of electrons and also undergo filamentation. Thus, as also clarified in their paper both in experimental observation and 2D PIC simulations, their parameter space lets them have near complete electron evacuation of their plasma channel by ponderomotive action of laser pulse leading to final coulomb explosion of the ion channel and ion acceleration (Maxwellian energy spectre) in their low density case; while in their high density case there is laser beam filamentation and subsequent shock acceleration of ions due to multiple filamentation interaction (Maxwellian spectre with plateau). So their maximum ion energy scaling is representative of their parameter space where Coulomb explosion of ion channel along with multiple filamentation assisted shocks govern ion acceleration process.

On the other hand we are in the totally different regime defined by: *τ*_*pi*_/*τ* (∈[26.74, 245.2] for the total scan and ∈[69.11, 245.2] for the scaling data) 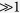, implying that ion dynamics within the pulse is unimportant; range of *λ*_*p*_ (∈[4.5, 42] *μm*) includes *cτ* (~10.5 *μm*) implying we scan over different electron acceleration regimes from bubble or blowout through resonant linear wake field acceleration (LWFA) to self modulated wake field acceleration (SMWF)^28^; *P*_*L*_/*P*_*c*_ ∈ [0.5, 50] implying that we are in the moderately relativistic regime but we do not undergo complete evacuation of the plasma channel and neither we observe any filamentation of the laser beam. The other parameter laser focal diameter to plasma wavelength ratio *d*_*μ*_/*λ*_*p*_ has similar values in both in ref. 10 and in our case. All these allow us to perform the experiment where we are able to be in a regime where maximum energy ions are coming from not the Coulomb explosion of the plasma channel, but from the pinching of much smaller size central ion filament at the gas exit end which is formed in the first place due to electron acceleration dynamics. Thus this scaling is in a new regime of ion acceleration representative of ultrashort pulse domain.

### Ion acceleration mechanism and directionality

The ion acceleration process under similar ultrashort laser conditions was previously studied in simulations in the ideal case of ultra-thin, infinitely sharp underdense *H* plasma box (~20*λ* × 20*λ* × 50*λ*)^13^. It was numerically possible to check ion response, although over very short time scales, and identify formation of a central ion filament inside the laser propagation channel under the blowout regime of electron acceleration. Subsequent expansion of this filament led to ion acceleration. These simulation conditions were too far from real experimental target conditions.

In order to explain the observed ion quasi monoenergetic features and their directionality we have carried out 3D PIC simulations in the case of our real full hyper-Gaussian He gas density profile and experimental focal spot configurations for much longer time scales up to 12 ps capturing full ion dynamics under our situation. We have been able to reproduce the non-Maxwellian ion energy features and explain the acceleration mechanism at *n*_*e*_ = 0.016 *n*_*c*_^18^. Unfortunately the cost of simulations prohibited us to carry out a scan in density. It is revealed that in this regime of interaction, different ion populations (Maxwellian and non-Maxwellian peaks) are accelerated through different mechanisms prevalent in different regions of the gas jet. The ion energy distribution has three populations. The very low energy part of the spectrum (not shown) is dominated by the radial part of the wake field^13^,^18^,^30^. The low energy branch in the ion spectra is created by an electrostatic shock around the expanding central ion filament, as seen in ref. 13. The energies coming from the mechanisms in ref. 13 are compatible with those of the observed low energy branch.

The highest energy branch, on the other hand, comes from an ion filament in the down ramp of the gas jet. In the falling density ramp of the plasma, local tenuous electron population is unable to screen the ion charge and a radially focusing and longitudinally accelerating electric field is initiated for several ps^18^. As the longitudinal plasma density gradient is not sharp enough (unlike in ref. 13), the radial field overtakes the longitudinal one. Thus the ions are pushed towards the laser propagation axis by the quasi-electrostatic field in the sheath and form a high density filament at the exit end of the gas jet which finally Coulomb explodes. The superposition of the longitudinal target normal sheath acceleration (TNSA) like field and the radial field coming from Coulomb explosion of the filament results in ion acceleration with an exit angle between 50°–80° as detected in the experiment. This is the reason why no ions are observed in the forward direction in TP2 for these interaction conditions.

The TNSA like contribution in this regime can be enhanced by either sharpening the gas density gradient^13^,^18^ or by increasing the hot electron current density^17^ making forward ion acceleration feasible. For example, use of gas mixture (high *Z* and low *Z* gases) in ref. 17 resulted in higher electron beam charge through ionization induced trapping allowing forward ion acceleration. Here the longitudinal electric field results from the magnetic field generated by the high return current^2^,^3^ due to the high charge content of the electron beam. However in the forward direction for pure *He* gas, in conformity with our observation, they also do not observe any ions beyond noise level.

### Variation of *E*_*C*_ with plasma electron density *n*_*e*_

We now discuss the physics of the dependence of the maximum ion energy upon the plasma density as presented in Fig. 3. The measured cut off energy *E*_*C*_ (Fig. 3), determined by the highest energy ions originating at the gas density down ramp as discussed above, thus depends on several factors: the formation of the central ion filament which depends on laser propagation across the plasma and subsequent electron acceleration; the strength of the sheath field which is related to the plasma gradient and the hot electron current; the sustenance of the sheath field in time^31^ which depends on the local azimuthal magnetic field dynamics^2^,^3^,^13^,^18^ and the efficiency of the self-pinching of the filament which depends on local electronic shielding^13^,^18^. Since we access a much wider range of plasma densities different electron acceleration mechanisms (Blowout, LWFA, SMWF etc.^28^) which control the hot electron current would play its role. The complete physics that quantitatively determines *E*_*C*_ over such a wide range of plasma densities is complex and beyond the scope of this experimental work where we present a simple discussion instead.

**Figure 5:**
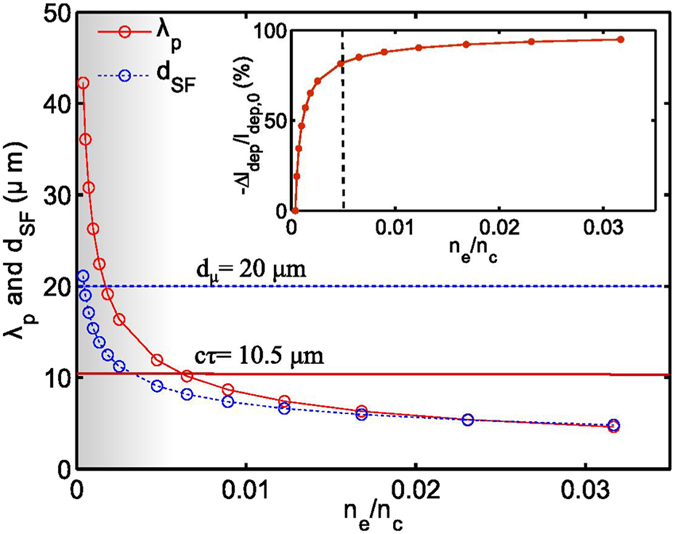
Estimated variation of plasma wavelength *λ*_*p*_ (dashed curve) and self focussed diameter *d*_*SF*_ (solid curve) with plasma electron density. For a given pulse intensity and plasma density the laser focal spot diameter *d*_*μ*_ (dashed line) and longitudinal pulse length *cτ* (solid line) sets the conditions of transverse wave breaking and bow wave generation. The inset shows that change in pulse depletion length *l*_*dep*_ with respect to initial value as a function of electron density.

The ion energy scaling (Fig. 3) as discussed before shows two distinct regions of interest: a density dependent sharp increase followed by a slowly varying part. To understand the scaling in Fig. 5 we plot *d*_*SF*_ and plasma wavelength *λp* ~ 2*πc*/*ω*_*pe*_ as a function of experimental plasma electron density *n*_*e*_. Figure 5 shows that the beam becomes more self focussed (the focussed beam diameter *d*_*SF*_ continues to reduce) with increasing density increasing the *in situ* intensity in the interaction region. Thus increasing the plasma electron density in this range is similar to enhancing the laser pulse intensity while still being in the blowout regime which is efficient for generating the central ion filament^13^ which Coulomb explodes at the falling density ramp to determine *E*_*C*_. Thus higher density in this range leads to stronger Coulomb explosion of the ion filament and hence higher *E*_*C*_. This determines the density dependent rise of *E*_*C*_ in Fig. 3. On the contrary in a regime where the pulse duration is larger than the inverse ion plasma frequency^8^,^10^, the fastest ions gain their radial momenta from Coulomb explosion, when the laser field is still present and bores a channel by expelling plasma electrons. They continue to drift undisturbed, due to their inertia, when the quasi-neutrality is restored when the electrons return, after the pulse is gone. Thus, in this regime, a denser plasma causes a stronger Coulomb explosion resulting in greater ion peak energies. This is different from the scaling obtained with a pulse duration shorter than the inverse ion plasma frequency.

The slowly varying part of *E*_*C*_ in Fig. 3 at comparatively higher plasma densities is modified under the combined effect of multiple processes: change in the nature of electron acceleration mechanism in this range of plasma densities^28^,^32^,^33^, laser propagation under the effect of pump depletion and ion charge neutralisation while traversing through neutral gas density^27^,^34^,^35^. The direct correlarion between electron acceleration and ion acceleration processes in this domain is a matter of further investigation. Laser propagation in this situation takes place in a density regime that satisfies the condition of bow wave generation^23^,^36^. When this condition is satisfied, bow wave is detached from the cavity generated by the laser field and this increase the electric potential of the wake wave^36^. The signature and strength of these effects is also encoded in the decrease in laser pulse energy depletion length, which under our experimental conditions is given by *l*_*dep*_ ~ *a*_*SF*_*cτ*(*n*_*c*_/*n*_*e*_)^36^. The inset in Fig. 5 shows that percentage change in pulse depletion length *l*_*dep*_ with respect to initial value *l*_*dep*,0_ as a function of electron density. We note that *l*_*dep*_ initially decreases rapidly with density and saturates around the density value (dashed line in inset) where the ion cut off energy also starts to saturate. The depleted laser energy goes into the plasma enhancing the wake potential and, under proper conditions, a component can also be converted into high harmonic radiations^4^,^37^. Another contribution to the slow decrease in the detected ion cut off would be that, a fraction of the accelerated energetic *He* ions would be neutralized because of large one^27^ and two^34^,^35^ electron capture crosssections at these energies that neutralizes the accelerated species before it reach the detector because they have to travel through denser neutral *He* gas. This possibly influences the diminishing trend in Fig. 4 for the total number of detected ions with plasma density. These could be identified by measuring the number of accelerated neutral atoms in such experiments^38^.

## Conclusions and Outlook

In conclusion, the measurement of ion acceleration over a wide range of plasma densities allowed us to identify a new acceleration regime. This experiment performed under these unexplored conditions enabling the identification of a new scaling of ion cut off energy with plasma electron density. Both *He* ion species have been detected in agreement with previous reports but for the first time, we demonstrate charge exchange between the ion species over the inspected density range. Our results shed light on the density dependence of the maximum ion energy in such experiments with underdense plasma. The interaction is clearly categorized into three different density regimes. We have identified a region in which the accelerated ion cut off energy increases sharply at low density, then reaches an optimum at about 0.5% *n*_*c*_ and finally decreases slowly. We have further provided a discussion of possible lines of theoretical and experimental work in this domain. Gas jet targets are debris free, easily customisable and reusable and make an attractive alternative test bed for ultra-short laser assisted ion acceleration schemes. Proper experimental optimisation of the longitudinal sheath field can be attained by tailoring the gas density gradient and/or through additional control of the gas density content e.g., utilising ionisation injection^39^ where for the same laser power, longitudinally accelerated electrons can reach higher energy enhancing the sheath field^31^,^40^. These possibilities open up the prospect of ion acceleration in this new intense ultra-short pulse domain motivated further by control of the cut off ion energy, ion populations and directions. In our view, this new evidences and subsequent discussions would motivate further experimental and theoretical investigations not only for fundamental laser plasma studies but also ion acceleration from gas jets at high repetition rate and in the ultrashort pulse domain.

## Additional Information

**How to cite this article**: Kahaly, S. *et al.* Detailed Experimental Study of Ion Acceleration by Interaction of an Ultra-Short Intense Laser with an Underdense Plasma. *Sci. Rep.* **6**, 31647; doi: 10.1038/srep31647 (2016).

## Figures and Tables

**Table 1:** Important parameters (first column), in addition to gas density profile, that affect ion acceleration in underdense plasma and the major sub-processes controlled by them (second column).

Parameters	Controlled sub-processes	Our case	Reference^10^
*τ*_*pi*_/*τ*	Ion dynamics within laser pulse duration	∈[26.74,245.2] » 1	∈[0.95, 5.6] ~ 1
*cτ*/*λ*_*p*_	Electron acceleration mechanisms^28^ and the subsequent electron energy distributions^32^,^33^	∈[0.3, 2.3] ~ 1(*cτ* ~ 10.5 *μm*; *λ*_*p*_ ∈ [4.5, 42] *μm*)	∈[10.9,64.3] » 1(*cτ* ~ 180 *μm*; *λ*_*p*_ ∈ [2.8, 16.5] *μm*)
*P*_*L*_/*P*_*c*_	Self-focusing^26^,^29^ and the degree of electron evacuation from the laser drilled plasma channel^10^,^18^	∈[0.5, 50]	∈[60, 2080]
*d*_*μ*_/*λ*_*p*_	Wake field curvature^28^,^32^,^41^ determining the radial field in the laser wake affecting electron and ion dynamics^13^,^16^,^18^	∈[0.5, 4.4]	∈[0.6, 3.6]

The operating parameter space in our case (third column) is compared with a previous experiment^10^ for which ion energy scaling is available (last column).
